# *Aspergillus* IgG antibody testing in the diagnosis of hypersensitivity pneumonitis: A scoping review

**DOI:** 10.1177/14799731251326592

**Published:** 2025-04-16

**Authors:** Lana Hatim, David W Denning

**Affiliations:** Faculty of Biology, Medicine and Health, 5292 The University of Manchester, Manchester Academic Health Science Centre, UK

**Keywords:** Extrinsic allergic alveolitis, ground glass, pulmonary fibrosis, occupation, fungal

## Abstract

**Background:**

Diagnosis of hypersensitivity pneumonitis (HP) or extrinsic allergic alveolitis requires a combination of tests with antibody testing playing a supportive role to identify exposures.

**Objectives:**

We conducted a scoping review on *Aspergillus* antibody testing in *Aspergillus*-related HP to identify the utility and diagnostic cutoffs proposed in the literature. We compared these cutoffs with studies of chronic pulmonary aspergillosis (CPA) and manufacturers’ cutoffs.

**Eligibility criteria:**

Only studies addressing the diagnostic value of *Aspergillus* IgG or precipitins for HP were included. Separately papers defining cutoffs for CPA were tabulated.

**Sources of evidence:**

Published papers were identified in literature searches in Embase, Web of Science, and Medline.

**Results:**

We identified 414 papers, of which 12 were included, all published between 1965 and 2005. Occupational HP linked to *Aspergillus* spp. exposure included Farmer’s Lung, Malt-Worker’s Lung, Esparto Worker’s Lung, and Woodworker’s lung (Sawmill-workers). No studies directly addressed serological testing in Tobacco Worker’s lung, Compost Lung, or poultry workers. Among *Aspergillus* species exposure, *A. fumigatus* was most commonly described; others included *A. umbrosus* (now *A. glaucus*), *A. clavatus*, and *A. niger*. Antibody tests included ELISA, BALISA, precipitin tests and ImmunoCAP, with a higher sensitivity of ELISA and ImmunoCAP tests compared to precipitin tests. Patients with HP linked to *Aspergillus* exposures, were positive in 156/290 (53.8%) compared to 96/615 (15.6%) in those with similar occupational exposures without HP. In malt workers with HP 35/53 (66%) had detectable *A. clavatus* IgG antibody compared to 0/53 *A. fumigatus* IgG, and 13/74 (18%) exposed but unaffected workers, but are not commercially available.

**Conclusions:**

Improved means of establishing or ruling out *Aspergillus* exposure are required, given the negative consequences for patients of continued *Aspergillus* inhalation. Modern studies with commercially available *Aspergillus* IgG antibody assays are required to define appropriate cutoffs for HP, given numerous studies published for chronic pulmonary aspergillosis.

## Introduction

Hypersensitivity pneumonitis (HP), also called extrinsic allergic alveolitis (EAA), develops when a susceptible individual repeatedly inhales various antigens (e.g. fungi, bacteria, avian proteins, or chemical source) resulting in inflammation of the lung parenchyma and smaller airways.^1^ HP was first described in 1713 by Bernardino Ramazzini in his book “*Diseases of Workers*,” where he described the condition in grain workers, or what is now known as Farmer’s Lung.^2,3^ In the UK, in 1932, Munro Campbell wrote the first clinical report of HP, documenting its occurrence in farmers exposed to mouldy hay.^3^ The first clinical report of HP in pigeon breeders was made in 1965 by Reed *et al.*, which is now known as Pigeon Breeder’s Lung, Bird Fancier’s Lung or Bird Breeder’s Lung.^3,4^ Today, more than 300 antigens, including *A. fumigatus* and various other microorganisms have been identified and found to be associated with HP.^5,6^

The ATS and ACCP guidelines (ATS/JRS/ALAT guideline 2020) categorize HP into non-fibrotic and fibrotic categories, based on the presence or absence of fibrosis, determined through radiological and histopathology results, which somewhat aligns with the disease outcome and prognosis.^7,8^ The clinical presentation of HP is variable, which complicates the diagnostic process; while some patients experience an acute presentation where symptoms improve within 24–48 h after exposure stops, others progress from non-fibrotic to fibrotic disease due to prolonged and repeated exposures to the inciting antigen.^9,10^ High resolution computed tomography scanning (HRCT) of the chest is the cornerstone of diagnosis and usually allows classification into non-fibrotic and fibrotic HP.^8,11^ Non-fibrotic HP presents with ground glass opacities, mosaic attenuations, and centrilobular nodules, alongside features like isolated air trapping, airspace consolidation, and lung cysts.^8,9^ Fibrotic HP presents additional features such as bronchiolar obstruction and lung fibrosis, sometimes with honeycombing.^8^ The presence of the ‘three density sign’ or ‘headcheese sign’ has a high specificity for fibrotic HP with a specificity of 93%.^10,12^ Confirmation and support of the diagnosis of HP comes from lung biopsy and a high lymphocyte count in bronchoalveolar lavage fluid in non-fibrotic HP.

The primary management requires removal of the patient from the source of antigen,^10,13^ so identifying the source is important and, given that most identified exposures are occupational, has important economic consequences for the patient. Antigen avoidance leads to improved lung function and longer survival, including transplanted patients.^14,15^ Patients with non-fibrotic HP tend to survive longer and have fewer recurrences when they avoid exposure to the antigen causing HP.^13^ However, identification of inciting antigen is not possible in majority of fibrotic HP patients.^8,16^ In many instances multiple antigens are responsible, with *Aspergillus* (if implicated) only one of the responsible exposures.

Numerous occupations and occupational diseases include or are driven by exposure to *Aspergillus* spp. including Wigmakers, Bird Fancier’s Lung, Compost Lung, Farmer’s Lung Disease, Malt-Worker’s Lung, Esparto Worker’s Lung (stipatosis), Tobacco-worker Lung, and Woodworker’s lung (Sawmill-workers), onion and potato exposure, and poultry workers.^17–32^ Home or activity-related *Aspergillus* exposures include duvets and pillows, air conditioning or humidifiers in homes, offices and vehicles, mould in damp homes or worker places and wind musical instruments.^33–38^ Given this wide range of possible exposures and the clinical importance of establishing the inciting antigens, IgG (precipitins) serology should be helpful.

Only some of the assays used in tracing the airborne exposures of patients with HP are commercially available, and in most cases the cutoff between positive and negative is based on 20–40 normal controls.^39,40^ Immunoglobulin G assays to *A. fumigatus* are widely available because of their utility for diagnosing chronic pulmonary aspergillosis and to a lesser extent *Aspergillus* rhinosinusitis, allergic bronchopulmonary aspergillosis, *Aspergillus* bronchitis and invasive aspergillosis in non-immunocompromised patients.^41–44^ Numerous assays in different formats are available, with different cut-offs, usually based on performance for chronic pulmonary aspergillosis, or a mixture of patients with aspergillosis. The most widely available assay, ImmunoCap^TM^, also provides assays for IgG directed against *A. niger* and *A. flavus*.^40^ We set out to survey the utility of *A. fumigatus* IgG assays in the diagnosis of HP and in particular to check if the cutoffs used for CPA diagnosis also may be applied to HP patients.

## Methods

In this study, a scoping review was conducted using the five-stage framework proposed by Hilary Arksey & Lisa O’Malley.^45,46^ Ethical approval was not required, as this scoping review examined existing published studies. A thorough literature search was performed using the databases Embase (Ovid), Web of Science, and Medline (Ovid). This search employed specific keywords (i.e. HP, extrinsic allergic alveolitis and the individual occupational disease terms) and subject-headings as listed in supplemental materials, with no restrictions on publication date to make sure all relevant studies are included; the searches were conducted on 11th July 2024 (see supplement for full search terms). Inclusion and exclusion criteria were established to select relevant studies. The inclusion criteria required that papers must (1) include a suitable control group for comparison; (2) include *Aspergillus* spp. in antibody test findings; and (3) be primary/original studies. The exclusion criteria were (1) lack of full text availability; (2) books, letters, poster/conference abstracts, case reports, reviews, meta-analyses, or conference papers; (3) animal studies; (4) non-English papers; (5) follow-up studies and (6) undefined or unspecific HP antigen exposure types. All studies from each database were exported to EndNote 20 for management and selection. The established eligibility criteria were applied to select relevant papers using the PRISMA flowchart guidelines Figure S1, by both authors (Supplemental material).^47^

A second thorough literature search in Medline and the Aspergillus Web site (https://www.aspergillus.org.uk/) was done to identify all the published cutoffs of *Aspergillus* IgG used for different commercial assays and different forms of aspergillosis, mostly chronic pulmonary aspergillosis.

The characteristics of the 12 included studies were individually tabulated. The characteristics of each study was analyzed using descriptive statistics, summarizing the major characteristic of the studies in the form of percentages. The studies were organized and reviewed according to different HP exposure types, and data from those with *A. fumigatus* or *A. clavatus* IgG cutoffs summed. No-meta analysis was conducted due to the different nature of the studies, including variations in diagnostic tests, antigens, environmental conditions, exposure types and control groups. Separately all studies describing a cutoff of *A. fumigatus* IgG for currently commercially available assays were tabulated.

## Results

Database searches resulted in a total of 779 papers of which 365 were duplicates leaving 414 papers for screening. Following title and abstract screening, 355 papers were excluded, resulting in 59 papers being selected for full-text review. After full-text review, 47 papers were excluded, resulting in 12 studies with original data utilising *Aspergillus* IgG testing for the diagnosis of HP (Figure S1).

The 12 studies included were cross-sectional studies published from 1965 to 2005. Collectively they reported on 554 patients with suspected or confirmed HP and 2576 controls without HP, including exposed and unexposed subjects, with or without other respiratory conditions (Table 1). Likely exposures for all HP cases included different bacteria and fungi; concerning *Aspergillus spp.*, *A. fumigatus* was most commonly described and other species included *A. umbrosus* (now synonomised with *A. glaucus*),^48^ *A. clavatus* and *A. niger*. These studies tested for *Aspergillus* IgG and other antigens using different antibody testing methods including ELISA, BALISA, precipitation tests and ImmunoCAP. Of these assays, only ImmunoCap is currently commercially available, although other *Aspergillus* IgG assays are sold, including immunoprecipitation assays (different from those published).

**Table 1.** Data chart depicting characteristics of included cross-sectional studies (*n* = 12).

Authors, year	Country	Aims	HP exposure type	Serological technique	Subject groups
^67^	U.K.	To investigate precipitin reactions in Farmer’s Lung patients, identify relevant antigens, compare serological techniques, and contrast results with other control groups, including other lung diseases	Farmer’s Lung	Double diffusion; Immuno-electrophoresis^67^	Study group: 1. Farmer’s Lung Disease - exposed to mouldy hay: *n* = 205 Control group: 2. Exposed to mouldy hay but do not have Farmer’s Lung Disease: *n* = 122 3. Non-farmers: *n* = 134
^68^	USA	To investigate serological reactivity and cross-reaction patterns observed in patients with various types of HP (including Farmer’s Lung Disease) and aspergillosis to different strains of *A. fumigatus*, 9 common fungi (*Mucor*, *Rhizopus, Cephalosporium*, *Fusarium*, *Penicillium*, *Alternaria*, *Hormodendrum*, *Candida albicans*, *Trichoderma*) and dusts	Farmer’s Lung; Pigeon breeder's disease; textile worker pneumonitis	Double diffusion agar gel, antigen production^69^	Study group: 1. Farmer’s Lung Disease: *n* = 20 2. Pigeon breeders disease: *n* = 19 3. Textile worker pneumonitis: *n* = 10 Control group: 4. Aspergillosis: *n* = 36 5. Healthy controls: *n* = 42 6. Asthma: *n* = 29
^70^	Finland	To investigate prevalence of antibodies to the antigens of A*. fumigatus*, *M. faeni*, and *T. vulgaris* in farmers, non-farmers, and respiratory patients (including those with Farmer’s Lung Disease)	Farmer’s Lung	Immunoprecipitation with modification^71^	Study group: 1. Patients (suspected of fungal allergy): *n* = 48; 1.1. Patients (Farmer’s Lung Disease): *n* = 22 Control group: 2. Farmers: *n* = 325 3. Non-farmers: *n* = 80
^72^	Finland	To investigate antibody levels to *A. fumigatus* among patients with Farmer’s Lung Disease, different pulmonary diseases, and healthy controls and to study the correlation between precipitin and ELISA tests	Farmer’s Lung	ELISA IgG	Study group: 1. Farmer’s Lung Disease: *n* = 31 Control group: 2. Miscellaneous respiratory diseases: *n* = 41 3. Bronchial asthma: *n* = 24 4. Non-allergic respiratory diseases: *n* = 16 5. Controls (healthy non-farmers: *n* = 63
^73^	Finland	To investigate role of antibody testing using ELISA in Farmer’s Lung Disease patients for the antigens of *A. fumigatus*, *T. vulgaris*, and *M. faeni*	Farmer’s Lung	ELISA IgG^74^	Study group: 1. Farmers’ Lung Disease patients: *n* = 17 Control group: 2. Bronchitis patients: *n* = 18 3. Controls (non-farmers, no respiratory symptoms): *n* = 20
^75^	Finland and USA (with contributions from Switzerland and Canada)	To measure the antibody levels against various HP antigens and strains (including *A. fumigatus*, *M. faeni, T. vulgaris, Penicillium, T. candidus, S. viridis, and A. umbrosus*) in patients with Farmer’s Lung Disease and control subjects from different countries	Farmer’s Lung	ELISA IgG^76 ^,^^^77^	Study group: 1. Farmer’s Lung Disease patients: *n* = 69 Control group: 2. Healthy lab employees: *n* = 28
^78^	Finland	To determine appropriate immunoglobulin class (IgG, IgA, IgM, and IgE) for diagnosing Farmer’s Lung Disease using ELISA focusing on antibodies against *A. umbrosus*, *A. fumigatus*, *T. vulgaris,* and *M. faeni*	Farmer’s Lung	ELISA IgG, IgA, IgM, and IgE^73^	Study group: 1. Farmer’s Lung Disease: *n* = 24 Control group: 2. Spouses of Farmer’s Lung Disease patients (exposed): *n* = 24;
^79^	Scotland	To investigate respiratory syndromes and the presence of precipitating antibodies against *P. granulatum*, *P. citrinum*, *R. stolonifer*, and *A. clavatus* in maltworkers	Malt-workers	Double diffusion agar gel^80^	Study group: 1. Maltmen (exposed): *n* = 114 Control group: 2. Carpet workers (dusty work): *n* = 25 3. Paper workers: *n* = 100
^81^	Scotland	To investigate maltworkers through environmental samples, sputum samples, prick tests and precipitins for fungal antigens. Precipitin test included antigens of *C. herbarum, A. niger, R. stolonifer, A. fumigatus, T. viride, A. clavatus* Also, relation between HP and antigens of *A. clavatus* was investigated	Malt-worker	Double diffusion agar gel, IE, immunoelectro-osmophoresis^67 ^,^^^82^	*Part 1:* Study group: 1. Maltworkers (exposed): *n* = 711 Control group: 2. Control sera: *n* = 50 *Part 2:* Study group: 1. Maltworkers suspected of having HP: *n* = 127
^83^	Spain	To investigate esparto grass exposure in symptomatic patients and measure antibody levels to *A. fumigatus, M. faeni,* and *T. vulgaris*	Esparto grass	Double diffusion, ELISA IgG^84 ^,^^^85^	Study group: 1. Esparto grass workers with suspected HP: (*n* = 5) Control group: 2. Healthy control sera: (*n* = 10)
^86^	Spain	To investigate IgG levels in workers with HP from esparto grass exposure and in healthy workers exposed to esparto, in response to *A. fumigatus*, *M. faeni,* and *T. vulgaris*	Esparto grass	Double diffusion and CAP IgG (Pharmacia, Upsala, Sweden)	Study group: 1. Esparto-grass HP workers : *n* = 5, Control group: 2. Healthy workers exposed to esparto: *n* = 45, 3. Unexposed controls *n* = 20
^22^	Norway	To investigate and compare exposure level, symptoms and IgG antibodies in two populations that each include wood-trimmers and planning operators	Wood-trimmer disease (Sawmill workers)	ELISA IgG^87^	Suspected wood trimmer disease cases): (total = 170) Wood trimmer: *n* = 99 Planning operator (control): *n* = 71 No disease control: Wood trimmers: *n* = 113 Planning operators (control): *n* = 190

ELISA: Enzyme-linked immunosorbent assay; IE: lmmunoelectrophoresis. CAP: ImmunoCap.

The studies included four from Finland all on Farmer’s Lung, two from Scotland on malt workers, two from Spain on Esparto grass workers, one from USA, one from both Finland and the USA with contributions from Switzerland and Canada, one from UK and one from Norway on sawmill workers (Table 1). Various occupational exposures were addressed, including six studies focused solely on Farmer’s Lung, one study addressing Farmer’s Lung along with other HP exposure types (textile worker pneumonitis and Pigeon Breeder’s disease), Malt Worker’s Lung, sawmill workers and stipatosis (esparto grass workers). No studies directly addressed serological testing in Tobacco Worker’s lung, Compost Lung, or poultry workers.

Exposures were usually to multiple antigens, including *A. fumigatus*, *A. umbrosus, A. clavatus* and *A. niger*. We know from more recent studies that detection of precipitating antibodies using the original Ouchterlony gel method, and its adaptations is less sensitive than the best ELISA and automated methods. The proportions of patients with HP, exposed but unaffected patients and non-diseased and unexposed controls are shown in Table 2.

**Table 2.** *Aspergillus fumigatus* antibody data from the HP studies where the positive and negative proportions are presented.

Occupation	Antibody detected/antibody negative (%)			Method	Reference
	HP	Exposed without HP	Other controls		
Farmers	63/166 (38%)	Healthy farmers 1/27 (4%)		Immunodiffusion	Pepys, 1965^67^
		Lung diseases 12/87 (14%)			
Farmers	13/20 (65%)	4/19 (19%)	Healthy 2/42 (4%)	Immunodiffusion	Flaherty, 1974^88^
		**–**	Asthma: 2/29 (7%)		
			Aspergillosis 30/36 (86%)^a^		
Farmers	18/22 (82%)	36/325 (11%)	3/80 (4%)	Immunoprecipitation	Katila, 1978^89^
Farmers	25/31 (81%)	19/41 (46%)	6/63 (10%)	ELISA	Mäntyjärvi, 1980^18^
Farmers	13/17 (76%)	4/18 (22%)	9/20 (45%)	ELISA	Ojanen, 1980^90^
Farmers	16/24 (67%)	4/24 (17%)		ELISA	Ojanen, 1992^91^
Malt-worker	0/53^b^	16/74 (22%)^c^	0/50	Immunoelectrophoresis	Blyth, 1977^81^
Esparto grass	5/5 (100%)		0/10	ELISA	Hinojosa, 1996^20^
Esparto grass	3/5 (60%)		7/20 (35%)	ImmunoCap	Gamboa, 2005^86^
**Totals** ^d^	**156/290 (53.8%)**	**96/615 (15.6%)**	**36/314 (11.1%)**		

^a^Omitted from total as a ‘positive control’

^b^Positives to *A. clavatus* antigen in those with HP was 35/53 (66%).

^c^Patients with other respiratory conditions, not HP.

^d^Omitting maltworker, given the lack of cross reactivity between *A, clavatus* and *A. fumigatus*.

Totalling all the patients described in papers with *A. fumigatus* serology with a defined cutoff (Table 2), 156/290 (53.8%) had a positive result. This contrasts with 96/615 (15.6%) exposed patients with evidence of HP in the same studies, and 36/314 (11.1%) apparently healthy, unexposed controls (although some controls were farmers). None of the malt workers with HP had a positive *A. fumigatus* IgG but 35 of 53 (66%) were positive to *A. clavatus* antigen, along with 13/74 (18%) exposed but unaffected workers. Only one of these studies used a commercial assay, ImmunoCap, and only five affected patients were included, all esparto grass workers.

In Table 3 are shown the published *A. fumigatus* antibody cutoffs, for all forms of aspergillosis, notably chronic pulmonary aspergillosis which has the highest fungal load in the lung for months or years. Fifteen studies addressed the cutoff for ImmunoCap, including two explicitly for HP patients. The cutoff values recommended for clinical reporting vary from 22 to 83mgA/mL, mostly derived from chronic pulmonary aspergillosis cases or healthy controls only, and conducted in several countries. Two studies from Germany focussed on HP patients utilising normal control sera recommended a cutoff of 65 mgA/L^40^ or 39 mgA/L (75% centile) or 78 mgA/L (90% centile)^39^ and one from Singapore reported a median *A. fumigatus* IgG of 31 mgA/L.^49^ Five studies recommended a cutoff for the Immulite 2000 assay of 10–20 mg/L, including one addressing HP patients, and *A. niger* IgG.^50–54^ The two studies with the Bordier *Aspergillus* IgG assay derived an optical density index of 0.82 or 0.9.^55,56^ The three Dynamiker studies derived cutoffs of 65-107AU/L.^50,53,57^ Only one study with data supporting a cutoff has been published for the BioRad Platelia assay,^58^ the Serion assay^50^ and the Omega (Genesis) assay.^50^

**Table 3.** *Aspergillus fumigatus* IgG cutoffs determined in the literature (2006 onwards).

Authors, year	Country	Assay	Patient group	Controls	Cutoff
Kranke, 2001^92^	Austria	ImmunoCap^a^	None	Healthy	39 mgA/L
Hoeyveld, 2006^60^	Belgium	Immunocap	None with HP	Healthy	35, 70^a^ mgA/L
Barton, 2008^93^	UK	ImmunoCap	Cystic fibrosis, ABPA, sensitised	Cystic fibrosis	90 mgA/L
Watkins, 2012^94^	South Africa	ImmunoCap	None	Healthy	67 mgA/L
Fujiuchi, 2016^95^	Japan	ImmunoCap	Untreated CPA	Chronic lung disease	50 mgA/L
Page, 2016^50^	UK	ImmunoCap	CPA	Healthy (Uganda)	20 mgA/L
Agarwal, 2017^41^	India	Immunocap	ABPA and allergic asthma	Healthy	27 mgA/L
Sehgal, 2018^62^	India	ImmunoCap	CPA	TB patients with CPA excluded	27 mgA/L
Al-Rahman, 2018^96^	Oman	ImmunoCap	None	Healthy	69 mgA/L^a^
Raulf, 2019^39^	Germany	ImmunoCap	None	Unexposed, healthy	78^b^, 97^c^ mgA/L
Tan, 2019^49^	Singapore	ImmunoCap	None	Healthy	31^d^ mgA/L
Huang, 2020^97^	Taiwan	ImmunoCap	CPA	Chronic lung disease	22 mgA/L
Lee, 2021^98^	Taiwan	ImmunoCap	CPA	Chronic lung disease, CPA excluded	40 mgA/L
Sennekamp, 2022^40^	Germany	ImmunoCap	Suspected HP	Healthy	64 mgA/L^e^
Hsiao, 2022^99^	Taiwan	ImmunoCap	CPA, IA and ABPA	Healthy	42 mgA/L
Salzer, 2023^63^	Multiple	ImmunoCap	Histologically proven CPA	Lung disease (including possible CPA) and healthy	83 mgA/L
Page, 2016^50^	UK	Immulite	CPA	Healthy (Uganda)	10 mg/L
Page, 2019^51^	Uganda	Immulite	TB and CPA patients	Healthy and screened controls with TB	20 mg/L
Jabeen, 2020^52^	Pakistan	Immulite	CPA	Chronic lung disease and healthy	20 mg/L
Setiangingrum, 2020^53^	Indonesia	Immulite	CPA	TB without CPA	11.5 mg/L
Intra, 2024^54^	italy	Immulite	HP	Healthy	10.2 mg/L
Shinfuku, 2023^58^	Japan	Platelia	CPA and ABPA	Colonised with *A. fumigatus*	16 AU/mL
Wilopo, 2020^55^	UK	Bordier	CPA	Healthy	0.9 ODI
Oladele, 2021^56^	Nigeria	Bordier	CPA	Healthy	0.82 ODI
Page, 2016^50^	UK	Serion	CPA	Healthy (Uganda)	35 U/L
Page, 2016^50^	UK	Dynamiker	CPA	Healthy (Uganda)	65 AU/L
Ma, 2019^57^	China	Dynamiker	IA and CPA	Healthy	89 AU/L
Setiangingrum, 2020^53^	Indonesia	Dynamiker	CPA	TB without CPA	107 AU/mL
Page, 2016^50^	UK	Genesis (Omega)	CPA	Healthy (Uganda)	20 U/L

CPA: chronic pulmonary aspergillosis; IA: invasive aspergillosis; ABPA: allergic bronchopulmonary aspergillosis; ODI: optical density index.

^a^97.5% centile.

^b^90% centile.

^c^95% centile.

^d^Median (SD = 31.7)

^e^The 90% centil was 146 mgA/L and the cutoff used in clinical work is derived from a prior paper.^59^

## Discussion

The first serum assays for exposure to *Aspergillus* and other antigens causing HP were precipitins assays. By diluting serum, a rough quantification of antibody titre could be determined. It was soon realised that not only was the titre variable in clear cut cases of HP, but also that some patients with HP did not have detectable precipitating antibody and that conversely that other exposed workers did without manifesting HP. Some laboratories do still run precipitins assays, often using double-diffusion immunoelectrophoresis procedures, but more automated assays have been adopted by most immunology laboratories these days, partly because of improved sensitivity.^60^ Themofisher/Phadia has a range of IgG assays in their ImmunoCap range including *Aspergillus fumigatus*, *A. flavus*, *A. niger*, budgerigar (GE90), pigeon (GE91) and *Micropolyspora faeni* (Gm22) (reclassified in 1989 as *Saccharopolyspora rectivirgula*). They do not offer a normal range for reporting *A. fumigatus* IgG declaring: “There is no common cut-off value for above normal levels of circulating specific IgG antibodies, as these are markers for allergen exposure, which may not be directly related to the disease and will depend on the local environment and levels of exposure”. Individual laboratories therefore have to decide their ‘normal’ range for the ImmunoCap assay, to issue reports to clinicians, based on internal validation or published work. Other manufacturers do recommend cutoffs for their assays.

In the last 10 years, many publications have emerged defining cut-offs for *A. fumi*gatus IgG in the diagnosis of chronic pulmonary aspergillosis (CPA) specifically. In this condition, the majority of patients have a positive *A. fumigatus* IgG, given the chronicity of the infection and huge microbial load of fungus in the lung. A key element of the design of these studies has been the control group, sometimes blood donors or surgical patients without lung disease, sometimes patients with other respiratory disorders, and sometimes both. Two German studies and one from Singapore, addressed the cutoff for possible HP using the ImmunoCap method. Monika Raulf and colleagues in Germany (2019) examined cutoffs for 32 antibody assays of possible antigens causing HP, using 121 sera from unexposed persons.^39^ For *A. fumigatus*, 75% of sera had antibody levels <39 mgA/L (90% centile was 78 mgA/L), and they proposed using an online calculator to define the cutoff. In Singapore, Yi Hern Tan et al found the median ImmunoCap *A. fumigatus* IgG antibody level in 120 healthy donors to be 30.9 +/− 31.7 mgA/L.^49^ Joachim Sennekamp and colleagues assessed 20 healthy persons and found 64 mgA/L to be the 95% in sera from 20 healthy donors and all 100 selected sera from possible HP patients to be above this.^40^ These cutoffs are higher than the cutoff value (40 mgA/L) currently used in the UK and other countries for diagnosing CPA,^61^ and higher than that recommended in India at 27 mgA/L.^62,63^ Jari Intra and colleagues in Italy (2024) compared 1850 sera from patients without HP to 54 with the disease linked to many different exposures using the Siemens Immulite 2000 assay for 6 antibodies including *A. fumigatus* and *A. niger*.^54^ The median and 95% centile of *A. fumigatus* IgG for controls and HP patients were 10.2 and 38.0 mg/L compared to 42.0 and 142.0 mg/L (*p* < 0.001). They also examined sera for antibody to *A. niger* with markedly different levels between controls and HP patients.

Some environmental exposures leading to HP are highly specific, including some chemicals and bird antigens. Others are more common, as for microorganisms found in hay (for many people an occasional exposure) or *Alternaria* linked to thunderstorms and wheat fields,^64–66^ as one example. But *Aspergillus* exposure is continuous of varying levels; for example compost and older pillows and bedding contain billions of spores. Occupational *Aspergillus* exposures clearly can lead to HP but may not; what is less clear is whether other non-occupational exposures may drive the onset of HP and/or pulmonary fibrosis. A combination of a clearcut diagnosis of HP, historical information (including occupation) indicating substantial fungal exposures and a positive *Aspergillus* IgG, is sufficient to infer (monovalent or polyvalent) causality. Unfortunately, pulmonary fibrosis may present without evidence of prior HP, specific exposure history is lacking and *Aspergillus* (or other fungal) IgG testing is negative. And this situation is further confounded by positive IgG antibody results in exposed persons without HP and several other forms of aspergillosis including allergic bronchopulmonary aspergillosis, with or without asthma, *Aspergillus* bronchitis or rhinosinusitis, *Aspergillus* nodules or chronic pulmonary aspergillosis. Additional work is required to address these uncertainties, especially better measures of exposure to specific antigens in those with lung fibrosis of uncertain aetiology. Given that antigen avoidance allows lung function to improve^14,15^ and that HP patients without pulmonary fibrosis survive longer and have fewer recurrences,^13^ redoubled efforts to address this area are required.

However, this scoping review has several limitations. A systematic quality appraisal of the selected studies was not conducted. Furthermore, due to variability in diagnostic methods and patient populations, a meta-analysis was not considered appropriate for these studies. As HP is not a commonly diagnosed disease, the data is limited to smaller studies, making it challenging for some studies to draw definite conclusions. Diagnostic criteria have been updated and changed, and previously differed between countries, which introduces inconsistency and complicates interpretation and comparison of older and newer study findings together. Some studies failed to mention HP exposure types or include appropriate controls for comparison, causing these to be excluded. The majority of studies on Farmer’s Lung Disease were from Finland, which may limit the findings to that country. No studies directly addressed serological testing in Tobacco Worker’s lung, Compost Lung, or poultry workers in which exposure to *Aspergillus* spp. is implicated. Our review was also limited to studies in English, which may have excluded additional findings.

In conclusion, we review the current understanding of how *Aspergillus* antibody testing could aid in the diagnosis of HP, with significant repeated exposure to *Aspergillus* spp. and contrast the cutoffs for *A. fumigatus* IgG antibody with other forms of aspergillosis. Unlike other HP exposures, which reflect antigens unrelated to other disorders, *Aspergillus* spp. is responsible for several different disease entities, with a corresponding rise in detectable *A. fumigatus* IgG. We highlight the difficulties in establishing cut-off values to differentiate between prior infection or exposure and disease. Recent studies have been published which aim to define cut-off values for specific IgG to *A. fumigatus* but further standardization is required to accommodate HP sera.

## Supplemental Material

**Supplemental Material -**Aspergillus IgG antibody testing in the diagnosis of hypersensitivity pneumonitis: A scoping review Supplemental Material for Aspergillus IgG antibody testing in the diagnosis of hypersensitivity pneumonitis: A scoping review by Lana Hatim and David W Denning in Chronic Respiratory Disease.
