# Supplementary material for: Aspergillus IgG antibody testing in the diagnosis of hypersensitivity pneumonitis: A scoping review
Source: Chron Respir Dis. 2025 Apr 16;22:14799731251326592. doi: 10.1177/14799731251326592 (PMC12033569; doi:10.1177/14799731251326592)
Supplement: Supplemental Material -Aspergillus IgG antibody testing in the diagnosis of hypersensitivity pneumonitis: A scoping review [file sj-pdf-1-crd-10.1177_14799731251326592.pdf]

Hatim and Denning

Supplementary materials

Figure S1: PRISMA flow diagram. Diagram as per PRISMA guidelines (Page et al., 2021).

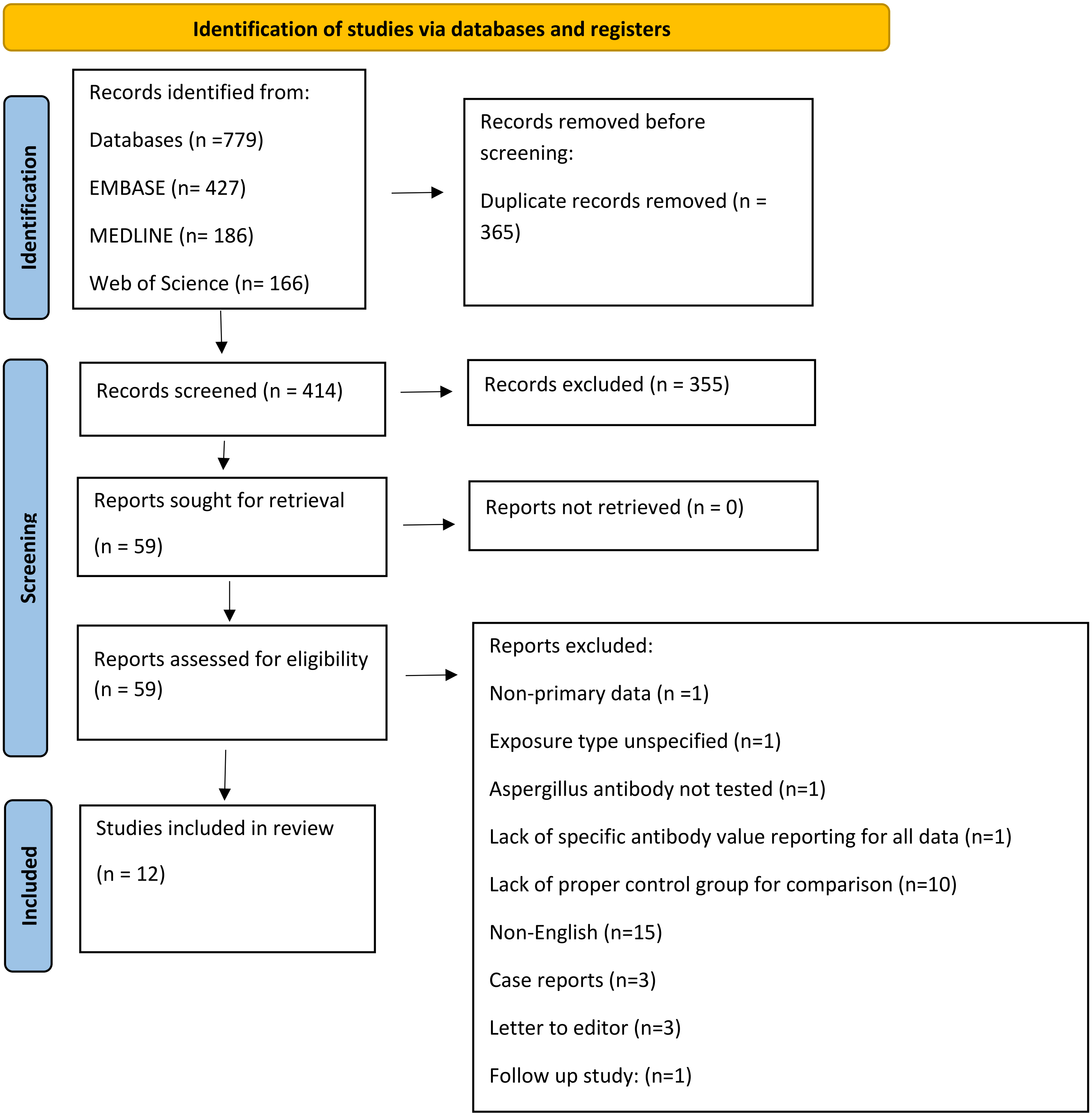

Table S1. Search strategy used in the selected databases.

Database	Search Strategy
**EMBASE**	**#1**. exp allergic pneumonitis/ OR "Hypersensitivity pneumonitis".ti,ab,kf. OR "Extrinsic allergic alveolitis".ti,ab,kf. OR "hypersensitivity pneumonia".ti,ab,kf. OR "Farmer's lung*".ti,ab,kf. OR "malt worker*".ti,ab,kf. OR "malt worker's lung".ti,ab,kf. OR"Esparto grass".ti,ab,kf. OR Esparto.ti,ab,kf. OR Compost.ti,ab,kf. OR Onion.ti,ab,kf. OR Potato.ti,ab,kf. OR Wood.ti,ab,kf. OR "Tobacco worker*".ti,ab,kf.<br><br>**#2**: exp Immunoglobulin/ OR Immunoglobulin*.ti,ab,kf. OR Antibod*.ti,ab,kf. OR IgG.ti,ab,kf. OR ELISA.ti,ab,kf. OR "enzyme-linked immunosorbent assay".ti,ab,kf. OR Precipitin*.ti,ab,kf. OR serolog*.ti,ab,kf.<br><br>**#3**: exp Aspergillus/ OR Aspergillus.mp.<br><br>**#4**: #1 AND #2 AND #3
**MEDLINE**	**#1:** exp Alveolitis, Extrinsic Allergic/ OR "Hypersensitivity pneumonitis".ti,ab,kf. OR "Extrinsic allergic alveolitis".ti,ab,kf. OR "hypersensitivity pneumonia".ti,ab,kf. OR "Farmer's lung*".ti,ab,kf. OR "malt worker*".ti,ab,kf. OR "malt worker's lung".ti,ab,kf. OR "Esparto grass".ti,ab,kf. OR Esparto.ti,ab,kf. OR Compost.ti,ab,kf. OR Onion.ti,ab,kf. OR Potato.ti,ab,kf. OR Wood.ti,ab,kf. OR "Tobacco worker*".ti,ab,kf.<br><br>**#2**: Immunoglobulin*.ti,ab,kf. OR Antibod*.ti,ab,kf. OR IgG.ti,ab,kf. OR

	ELISA.ti,ab,kf. OR "enzyme-linked immunosorbent assay".ti,ab,kf. OR Precipitin*.ti,ab,kf. OR serolog*.ti,ab,kf.<br><br>**#3**: exp Aspergillus/ OR Aspergillus.mp.<br><br>**#4**: #1 AND #2 AND #3
**Web of Science**	TS=(("hypersensitivity pneumonitis" OR "Extrinsic allergic alveolitis" OR "hypersensitivity pneumonia" OR “Farmer’s lung*” OR “malt worker*” OR “malt-worker's lung” OR “Esparto grass” OR “Esparto” OR “Compost” OR “Onion” OR “potato” OR “Wood” OR “Tobacco worker*") AND (Immunoglobulin* OR Antibod* OR IgG OR ELISA OR “enzyme-linked immunosorbent assay*” OR Precipitin* OR serolog*)) AND ALL=(Aspergillus)

Search strategy for Aspergillus IgG cutoff

“Aspergillus IgG” with restrictions of human only, and from January 1st 1980 onwards. No language restriction was applied.

All abstracts were screened for numerical values indication and comparison between a disease group and a control group.

Full text was then reviewed to identify if a the cutoff was derived by the experimental design, or arbitrarily selected – the latter papers were not included.
